# Prediction of Prognostic Factors and Genotypes in Patients With Breast Cancer Using Multiple Mathematical Models of MR Diffusion Imaging

**DOI:** 10.3389/fonc.2022.825264

**Published:** 2022-01-31

**Authors:** Weiwei Wang, Xindong Zhang, Laimin Zhu, Yueqin Chen, Weiqiang Dou, Fan Zhao, Zhe Zhou, Zhanguo Sun

**Affiliations:** ^1^ Department of Medical Imaging, Affiliated Hospital of Jining Medical University, Jining, China; ^2^ MR Research, GE Healthcare, Beijing, China

**Keywords:** breast neoplasms, magnetic resonance imaging, diffusion-weighted imaging, intravoxel incoherent motion, diffusion kurtosis imaging

## Abstract

**Purpose:**

To explore the clinical value of apparent diffusion coefficient (ADC), intravoxel incoherent motion (IVIM), and diffusion kurtosis imaging (DKI) based on diffusion-weighted MRI (DW-MRI) for predicting genotypes and prognostic factors of breast cancer.

**Materials and Methods:**

A total of 227 patients with breast cancer confirmed by pathology were reviewed retrospectively. Diffusion-weighted imaging (DWI), IVIM, and DKI were performed in all patients. The corresponding ADC, true diffusion coefficient (D), perfusion-related diffusion coefficient (D*), perfusion fraction (f), mean diffusion rate (MD), and mean kurtosis value (MK) were measured. Multivariate logistic regression analysis and receiver operating characteristic (ROC) curve were used to analyze the diagnostic efficacy in predicting the Nottingham prognostic index (NPI), the expression of antigen Ki-67, and the molecular subtypes of breast cancer. The nomogram of the combined genotype-prediction model was established based on the multivariate logistic regression model results.

**Results:**

D* and MK values were significantly higher in the high-grade Nottingham group (NPI ≥ 3.4) than the low-grade Nottingham group (NPI < 3.4) (p < 0.01). When D* ≥ 30.95 × 10^−3^ mm^2^/s and MK ≥ 0.69, the NPI tended to be high grade (with areas under the curve (AUCs) of 0.712 and 0.647, respectively). The combination of D* and MK demonstrated the highest AUC of 0.734 in grading NPI with sensitivity and accuracy of 71.7% and 77.1%, respectively. Additionally, higher D*, f, and MK and lower ADC and D values were observed in the high Ki-67 than low Ki-67 expression groups (p < 0.05). The AUC of the combined model (D + D* + f + MK) was 0.755, being significantly higher than that of single parameters (Z = 2.770~3.244, p = 0.001~0.006) in distinguishing high from low Ki-67 expression. D* and f values in the Luminal A subtype were significantly lower than in other subtypes (p < 0.05). Luminal B showed decreased D value compared with other subtypes (p < 0.05). The HER-2-positive subtype demonstrated increased ADC values compared with the Luminal B subtype (p < 0.05). Luminal A/B showed significantly lower D, D*, MD, and MK than the non-Luminal subtypes (p < 0.05). The combined model (D + D* + MD + MK) showed an AUC of 0.830 in diagnosing the Luminal and non-Luminal subtypes, which is significantly higher than that of a single parameter (Z = 3.273~4.440, p < 0.01). f ≥ 54.30% [odds ratio (OR) = 1.038, p < 0.001] and MK ≥ 0.68 (OR = 24.745, p = 0.012) were found to be significant predictors of triple-negative subtypes. The combination of f and MK values demonstrated superior diagnostic performance with AUC, sensitivity, specificity, and accuracy of 0.756, 67.5%, 77.5%, and 82.4%, respectively. Moreover, as shown in the calibration curve, strong agreements were observed between nomogram prediction probability and actual findings in the prediction of genotypes (p = 0.22, 0.74).

**Conclusion:**

DWI, IVIM, and DKI, as MR diffusion imaging techniques with different mathematical models showed potential to identify the prognosis and genotype of breast cancer. In addition, the combination of these three models can improve the diagnostic efficiency and thus may contribute to opting for an appropriate therapeutic approach in clinic treatment.

## Introduction

Breast cancer is the most common malignancy among women (1). The management and overall survival of breast cancer are highly individualized and routinely based upon prognostic factors, such as the Nottingham prognostic index (NPI), the antigen Ki-67, and molecular expression signatures (2, 3). The NPI is the most validated system in breast cancer with the least interobserver variability currently (4). A higher Nottingham grade is associated with shorter survival and early recurrence, irrespective of tumor size, hormone receptor status, or lymph node metastasis status (5, 6). The Ki-67 index, reflecting the extent of proliferative activity, is a reliable identifier of more aggressive breast cancer and is associated with high risk for metastasis or recurrence, worse prognosis, and decreased survival (7). Furthermore, preoperative genotyping of breast cancer is essential because it may predict neoadjuvant chemotherapy responsiveness and allow optimized strategies for patient-tailored therapy. The Luminal A subtype is less responsive to chemotherapy, whereas the Luminal B subtype is responsive not only to chemotherapy but also to endocrine treatment or molecular-targeted therapy. The HER-2-positive subtype is insensitive to endocrine therapy but sensitive to targeted drugs such as trastuzumab therapy (8, 9). Triple-negative breast cancer (TNBC) lacks expressions of all three receptors (ER, PR, and HER-2) and is known to have a more aggressive clinical course and poorer outcomes (10, 11). However, both the prognostic factors and genotypes need to be obtained by biopsy or surgery.

MRI has a greater sensitivity than mammography or ultrasound in the diagnosis of breast cancer (12). Both dynamic contrast-enhanced MRI (DCE-MRI) and diffusion-weighted imaging (DWI) can detect the microscopic features of tumors. However, DCE-MRI requires intravenous contrast media administration; thus, it is not suitable to be used in patients with renal dysfunction. Moreover, as a semiquantitative analysis, time-signal intensity curve (TIC) assessment was reported with a low specificity in benign and malignant breast lesions (13). DWI with apparent diffusion coefficient (ADC) is routinely used in breast diagnosis, but the reported diagnostic reliability is still controversial, mainly due to inaccurate depiction of water molecule diffusion with the Gaussian model and influence of microcirculation perfusion (14). To address these two issues, an extended diffusion model of diffusion kurtosis imaging (DKI) reflects non-Gaussian diffusive motions of water in biologic tissues and has the potential to characterize the tissue heterogeneity and the interaction between water molecules and adjacent tissues (15). Meanwhile, intravoxel incoherent motion (IVIM) with multiple b-values, as another advanced diffusion model, allows the usage of a bi-exponential model to derive fast and slow diffusion parameters, aiming to separate diffusion from perfusion behaviors and better reflect the internal situation of tumors (16).

So far, multiple diffusion imaging techniques, including DWI, IVIM, or DKI, have been applied to evaluate the diagnostic value for prognostic factors and genotypes previously (17–19). However, to our best knowledge, no study was implemented to systematically apply these three techniques with individual mathematical models for comparison and to investigate the potential of the combined model in discriminating the prognostic factors and genotypes of breast cancer.

This study aimed to quantitatively compare the diagnostic performance of DWI, IVIM, DKI, and combined models for discriminating the prognostic factors and genotypes of breast cancer.

## Materials and Methods

### Patients

**Figure 1:**
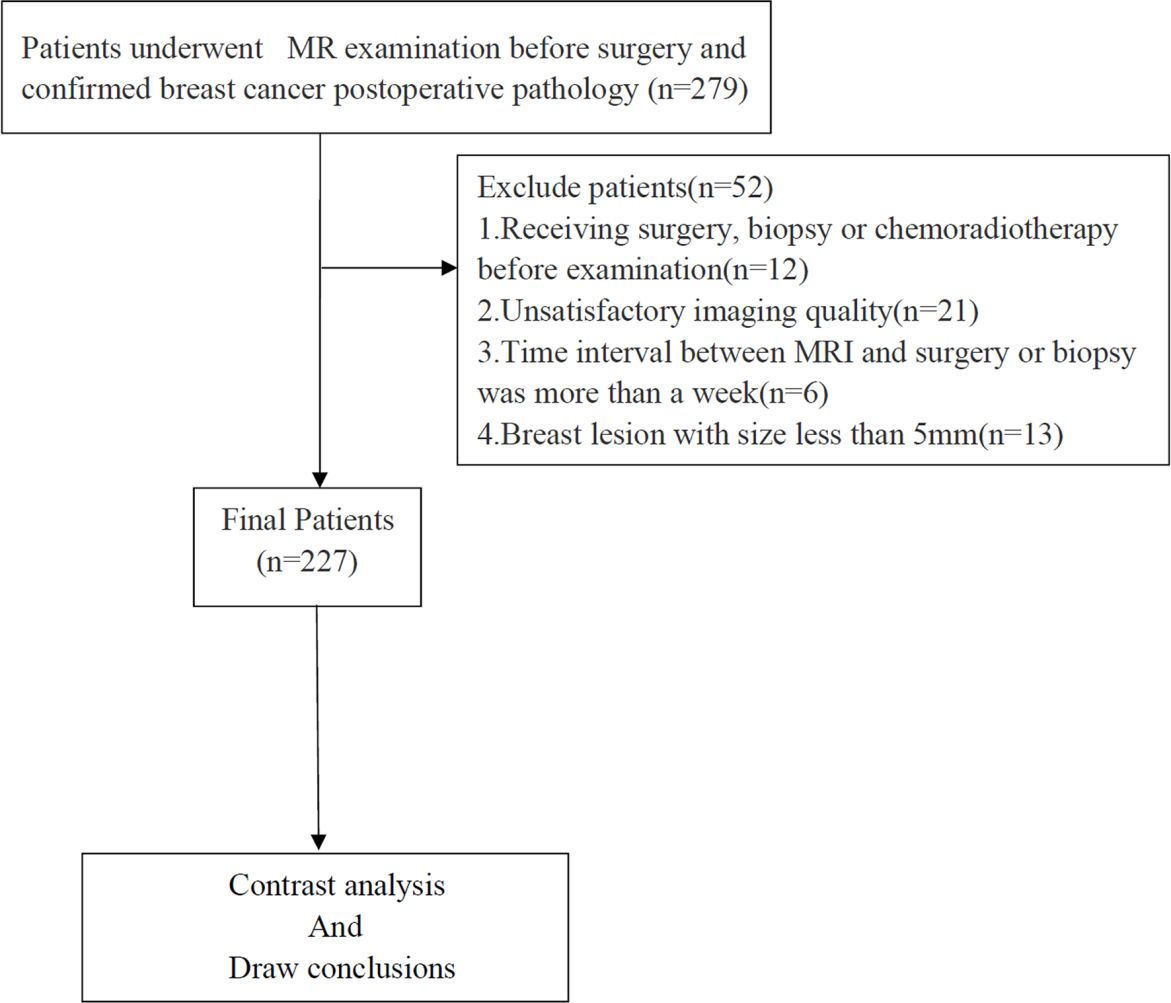
Flowchart of the enrolled patients.

From January 2019 to August 2021, 279 patients with breast cancer, confirmed by pathological examination, were recruited. The inclusion criteria were as follows: a) no contraindications to MRI examination; and b) all patients underwent routine MRI and multi-b-value DWI images. The exclusion criteria were as follows: a) receiving surgery, biopsy, or chemoradiotherapy before the examination; b) unsatisfactory imaging quality; c) time interval between MRI and surgery or biopsy was more than 2 weeks; and d) breast lesion with a size of less than 5 mm. **Figure 1** shows the flow diagram of the recruitment process. Finally, 227 patients were included in this study.

### MRI Acquisition

A 3.0-T MR scanner (Discovery MR 750W, GE Medical Systems, Chicago, IL, USA) with a 16-channel phased-array coil specific for breast imaging was used for all MRI experiments. All patients were scanned in the prone position, with breasts naturally suspended in the coil. Premenopausal patients were examined in the second week of the menstrual cycle. The routine scan sequences were performed as follows: a transverse axial fast spin-echo T1-weighted imaging (FSE-T1WI) sequence [repetition time (TR)/echo time (TE) = 420/10 ms, field of view (FOV) = 320 mm × 288 mm, slice thickness/gap = 5/1 mm] and an axial fat-suppressed fast-recovery fast spin-echo T2-weighted imaging (FRFSE-T2WI) sequence (TR/TE = 6,000/88 ms, FOV = 320 × 288 mm, slice thickness/gap = 5/1 mm). DWI, IVIM, and DKI were acquired before contrast injection using spin-echo echo-planar imaging sequence (SE-EPI). The parameters of DWI were as follows: TR/TE = 3,600/73 ms, the excitations (NEX) = 2, and b-values of 0 and 1,000 s/mm^2^. IVIM was performed with the following parameters: TR/TE = 2,500/90 ms, matrix = 128 × 128 mm. Thirteen b-values (0, 20, 30, 50,70, 100, 150, 200, 500, 700, 1,000, 1,500, 2,000 s/mm^2^) were used in three orthogonal directions. As the b-value increased, the number of NEX also increased from 1 to 6 to ensure a sufficient image signal-to-noise ratio (SNR). The total acquisition time for IVIM was 6 min 40 s. DKI was obtained at b-values of 0, 1,000, and 2,000 s/mm^2^. Fifteen diffusion gradient directions were set separately at b-values of 1,000 and 2,000 s/mm^2^. Other scan parameters were TR/TE = 5,000/90 ms, matrix = 128 × 128, NEX = 2, and scan time = 5 min 55 s. The section thickness/gap and FOV of DWI, IVIM, and DKI were copied from the FRFSE-T2WI sequence.

### Data Analysis

All images were transferred to Advantage Workstation (version AW 4.6, GE Medical Systems) for post-processing. Acquired DWI, IVIM, and DKI data were processed by vendor-provided software (Function tool MADC and DKI software; GE Healthcare) to acquire corresponding parametric maps.

For DWI, ADC maps were generated on a pixel-by-pixel basis according to a mono-exponential model: S_b_/S_0_ = exp (−b·ADC), where b is the diffusion factor, and S_b_ and S_0_ are the signal intensities with diffusion factors of 1,000 and 0 s/mm^2^ (20).

IVIM-derived parameters were calculated based on the following bi-exponential model: S_b_/S_0_ = (1 − f) × exp(−b × D) + f × exp[−b × (D* + D)], where S_b_ is the diffusion-weighted signal at a certain b-value; S_0_ is the signal without diffusion weighting at b = 0; D, true diffusion coefficient, represents pure water molecular diffusion in tissues; D*, pseudo-diffusion coefficient, a fast component of diffusion, reflects the incoherent movements of microvascular blood within the voxel; and f, perfusion fraction, represents the volume fraction of random microcirculation over the total incoherent signal in each voxel (21).

DKI parameters were calculated using the following equation: S_b_ = S_0_·exp(−b^2^·D^2^ + b·D^2^·K/6), where S_0_ and S_b_ represent the signal intensity (SI) under different b-values (0 s/mm^2^ or other values); K (arbitrary units) indicates kurtosis and represents the degree of deviation from the Gaussian distribution; and D (×10^−3^ mm^2^/s) indicates diffusivity and represents the diffusion coefficient corrected for non-Gaussian bias (22).

**Figure 2:**
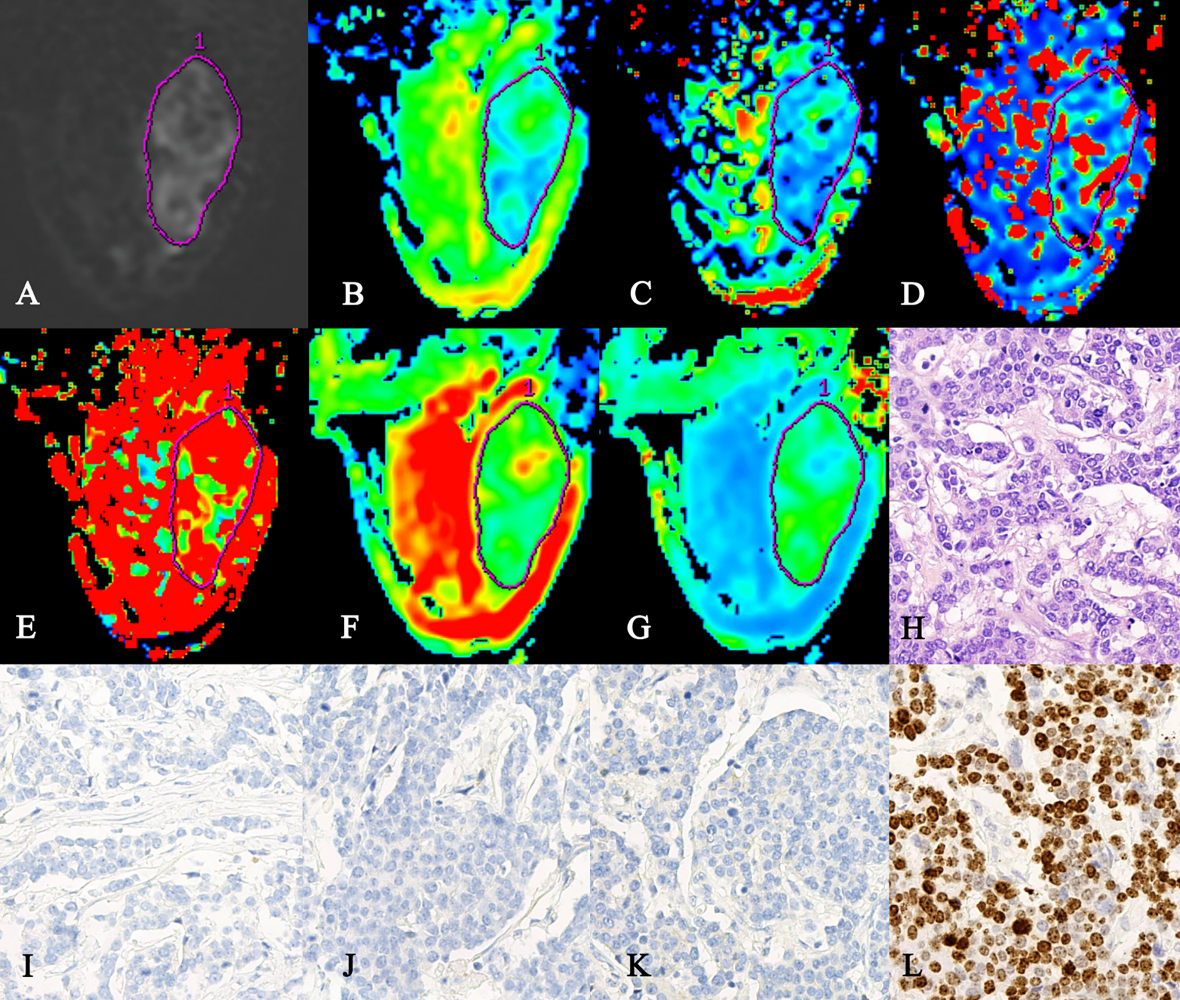
A 47-year-old female patient with triple-negative cancer in the right breast. **(A)** ROI was set on the grayscale map with b-value of 1,000 s/mm^2^. **(B–G)** The pseudo-colored maps of the ADC, D, D*, f, MD, and MK. ADC = 1.05 × 10^−3^ mm^2^/s, D = 0.92 × 10^−3^ mm^2^/s, D* = 41.8 × 10^−3^ mm^2^/s, f = 81.1%, MD = 2.58 × 10^−3^ mm^2^/s, and MK = 0.774. **(H)** H&E staining of the invasive breast ductal carcinoma (×200). **(I–L)** Immunohistochemistry staining for the ER **(I)**, PR **(J)**, HER-2 **(K)**, and Ki-67 **(L)** in the invasive breast ductal carcinoma. ROI, region of interest; ER, estrogen receptor; PR, progesterone receptor.

The region of interest (ROI) was delineated on the grayscale map with a b-value of 1,000 s/mm^2^ (12), and then, the pseudo-color images of the IVIM and DKI parameters were merged with the grayscale map by using 3D SynchroView (GE Healthcare) (**Figure 2**). The ROI included as much of the solid region of the tumor as possible, while regions with large blood vessels, necrosis, or hemorrhage were avoided. For patients with multicentric or multifocal tumors, only the tumors with the largest diameter were analyzed. For the non-mass lesions, the ROI was placed on the representative solid slice of the tumor by using the plain scan and contrast-enhanced sequence as references. The ROI was delineated by two independent radiologists (LZ and WW with 15 and 5 years of experience, respectively). In order to reduce the measurement error caused by the bias of ROI selection, the maximum layer of the lesion, and the upper and lower consecutive levels were measured three times. Then, the corresponding average value was calculated for data analysis.

### Pathological Evaluation

All patients underwent breast-conserving surgery or mastectomy, while 64 patients received additional biopsy before operation. Tumor size, axillary node status, histologic type, histologic grade, and lymphovascular invasion status were determined based on surgically excised specimens. The pathological evaluation of the surgically resected specimens was performed by two pathologists (with 4 and 12 years of experience) independently. NPI was calculated according to the following formula: NPI = size (cm) × 0.2 + lymph node staging (1–3) + histologic grade (1–3). No metastatic lymph nodes is 1 point, 1–4 nodes is 2 points, and more than 4 nodes is 3 points. Based on NPI scores, the low-grade (2.0–3.4 points) and high-grade (>3.41 points) groups were defined (23). Tumor subtypes were classified as Luminal A for ER or PR positive, HER-2 negative, and Ki-67 negative; as Luminal B for ER or PR positive, HER-2 positive, and Ki-67 positive; as HER-2 positive for ER and PR negative and HER-2 positive; and as triple negative (TN) for ER, PR, and HER-2 negative. ER and PR positivity were defined as 10% or with more positively stained nuclei in 10 high-power fields. Ki-67 labeling was defined as negative (<14%) or positive (≥14%). The intensity of HER-2 staining was semiquantitatively scored as 0, 1+, 2+, or 3+. Tumors with a 3+ score were classified as HER-2 positive, and tumors with 0 or 1+ score were classified as HER-2 negative. In tumors with a 2+ score, gene amplification with fluorescence *in situ* hybridization was used to determine HER-2 status (24).

### Statistical Analysis

Statistical analyses were conducted using SPSS 25.0 (IBM Corporation, Armonk, NY, USA), MedCalc 19.5.1 (Ostend, Belgium), and R version 4.0.0 (http://www.r-project.org/). The interobserver consistency was assessed by inter-class correlation coefficients (ICCs). The interpretation of ICC values was defined as follows: 0.00–0.20, poor agreement; 0.21–0.40, fair agreement; 0.41–0.60, moderate agreement; 0.61–0.80, good agreement; and 0.81–1.00, excellent agreement (25). The Kolmogorov–Smirnov test was used to determine whether two samples of measurement data were normally distributed. An independent-samples t-test was used to compare the ADC, D, MD, and MK values between different prognostic factors. The Mann–Whitney U test was used to detect the difference in D* and f values between the different prognostic factors. Moreover, one-way ANOVA was used for multiple comparisons of ADC, D, MD, and MK values between different genotypes; and the Kruskal–Wallis H test was performed to compare D* values among different genotypes. Multivariate logistic regression analyses were used to identify independent factors. Receiver operating characteristic (ROC) curves were used to assess the diagnostic efficacy of each parameter or model in discriminating prognostic factors or genotypes, and the Delong test was used to determine whether the area under the curve (AUC) of each ROC was significantly different. p < 0.05 was considered statistically significant. A nomogram was developed based on the outcomes of multivariate logistic regression to predict the genotypes. And a calibration using bootstraps with 1,000 resamples for internal validation by comparing nomogram-predicted versus nomogram-observed response probability was done as well as the Hosmer–Lemeshow goodness-of-fit test.

## Results

### Clinical and Pathological Characteristics

The average age of the 227 patients was 50.8 ± 10.3 years (range 27–86). The histological types included 206 invasive ductal carcinomas (90.7%), 7 ductal carcinoma *in situ* (3.1%), 4 invasive lobular carcinomas (1.8%), 3 mucinous carcinomas (1.3%), 3 invasive ropapillary carcinomas (1.3%), 3 medullary carcinoma (1.3%), and 1 cribriform carcinoma (0.5%). Of the 227 lesions, 151 (66.5%) were classified as Luminal subtype, 36 (15.9%) as HER-2-positive subtype, and 40 (17.6%) as TNBC subtype (**Table 1**).

**Table 1:** Clinicopathological characteristics of the included patients.

Characteristics	Data
**Age (years), mean ± SD**	50.8 ± 10.3
**Mean tumor size (cm), mean ± SD**	2.56 ± 1.22
**Histologic grade, n (%)**	
1	16 (7.0%)
2	99 (43.6%)
3	112 (49.4%)
**Histological type, n (%)**	
Invasive ductal carcinoma	206 (90.7%)
Non-invasive ductal carcinoma	21 (9.3%)
**Nodal status, n (%)**	
Negative (−)	95 (41.9%)
Positive (+)	132 (58.1%)
** ER, n (%)**	
Negative (−)	77 (33.9%)
Positive (+)	150 (66.1%)
** PR, n (%)**	
Negative (−)	95 (41.9%)
Positive (+)	132 (58.1%)
**HER-2, n (%)**	
Negative (−)	107 (47.1%)
Positive (+)	120 (52.9%)
**Ki-67, n (%)**	
Negative (−)	61 (26.9%)
Positive (+)	166 (73.1%)
**Genotypes, n (%)**	
Luminal A	29 (12.8%)
Luminal B	122 (53.7%)
HER-2-positive	36 (15.9%)
Triple-negative	40 (17.6%)

ER, estrogen receptor; PR, progesterone receptor.

### Interobserver Agreement

The ICCs between the two radiologists were 0.878 [95% CI: 0.842–0.906], 0.820 (95% CI: 0.766–0.861), 0.908 (95% CI: 0.880–0.929), 0.892 (95% CI: 0.860–0.917), 0.870 (95% CI: 0.832–0.900), and 0.886 (95% CI: 0.852–0.912) for ADC, D, D*, f, MD, and MK measurements, respectively, indicating an excellent interobserver agreement.

### Diagnostic Performance of Diffusion-Weighted Imaging-, Intravoxel Incoherent Motion-, and Diffusion Kurtosis Imaging-Derived Parameters in Differentiating Prognostic Factors of Breast Cancer

The D* and MK values were significantly higher in tumors of the high-grade Nottingham group (NPI ≥ 3.4) than those of the low-grade Nottingham group (NPI < 3.4) (p < 0.01). The D*, f, and MK values were higher and the ADC and D values were lower in the high Ki-67 expression group than in the low expression group (p < 0.05) (**Table 2**).

**Table 2:** Diagnostic performance of DWI, DKI, and IVIM parameters in different prognostic factors of breast cancer.

Parameters	NPI		p-Value	Ki-67		p-Value
	High	Low		<14%	≥14%	
ADC (×10^−3^ mm^2^/s)	0.99 ± 0.35	1.00 ± 0.33	0.884	1.08 ± 0.34	0.96 ± 0.34	0.025
D (×10^−3^ mm^2^/s)	0.67 ± 0.32	0.71 ± 0.33	0.407	0.76 ± 0.29	0.65 ± 0.33	0.022
D* (×10^−3^ mm^2^/s)	39.98 ± 26.22	23.89 ± 16.99	<0.001*	26.77 ± 15.97	39.70 ± 27.30	<0.001*
f (%)	40.17 ± 16.05	37.93 ± 14.77	0.346*	34.77 ± 14.18	41.44 ± 15.97	0.003*
MD (×10^−3^ mm^2^/s)	2.43 ± 0.76	2.45 ± 0.74	0.868	2.46 ± 0.79	2.39 ± 0.66	0.500
MK	0.74 ± 0.23	0.63 ± 0.19	0.001	0.63 ± 0.17	0.75 ± 0.23	<0.001

NPI, Nottingham prognostic index; DWI, diffusion-weighted imaging; DKI, diffusion kurtosis imaging; IVIM, intravoxel incoherent motion.

*Mann–Whitney U test.

### Diagnostic Efficiency of Diffusion-Weighted Imaging-, Intravoxel Incoherent Motion-, and Diffusion Kurtosis Imaging-Derived Parameters for Predicting Nottingham Prognostic Index and Ki-67

**Figure 3:**
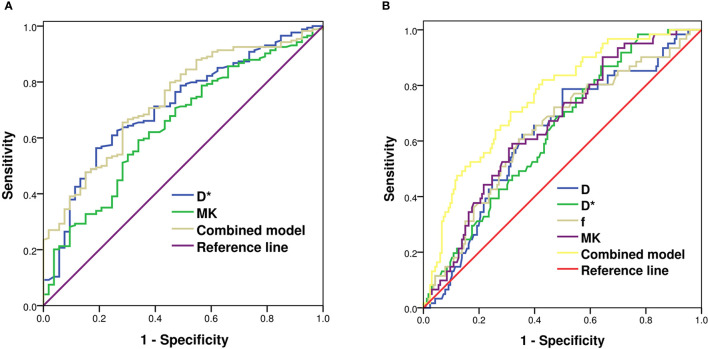
ROC analysis of IVIM and DKI parameters in predicting Nottingham index **(A)** and Ki-67 **(B)** of breast cancer. ROC, receiver operating characteristic; IVIM, intravoxel incoherent motion; DKI, diffusion kurtosis imaging.

According to the multivariate logistic regression, D* [odds ratio (OR) = 1.038, p < 0.001] and MK (OR = 24.745, p = 0.012) were found to be significant predictors of NPI. When D* ≥ 30.95 × 10^−3^ mm^2^/s and MK ≥ 0.69, the NPI tended to be high grade, and the AUCs were 0.712 and 0.647, respectively. The combination of MK and D* demonstrated the highest sensitivity and accuracy of 71.7% and 77.1%, respectively. The AUC of the combined model (D* + MK) was significantly higher than that of MK (Z = 2.148, p = 0.032), whereas there was no statistically significant difference from that of D* (Z = 0.879, p = 0.379) (**Table 3** and **Figure 3A**).

**Table 3:** Diagnostic efficiency of the IVIM and DKI models for predicting Nottingham index and Ki-67.

Parameters	AUC	95% CI	Cutoff	Sensitivity (%)	Specificity (%)	Accuracy (%)
**Nottingham index**						
D*	0.712	0.635~0.789	30.95 × 10^−3^ mm^2^/s	64.9	81.1	76.7
MK	0.647	0.563~0.730	0.69	59.2	66.0	73.2
Combined model	0.734	0.672~0.791	:	71.7	65.5	77.1
**Ki-67**						
D	0.625	0.545~0.705	0.68 × 10^−3^ mm^2^/s	60.7	65.7	71.8
D*	0.634	0.558~0.710	31.02 × 10^−3^ mm^2^/s	61.8	68.9	72.6
f	0.638	0.813~0.913	34.75%	65.7	60.7	73.1
MK	0.657	0.581~0.733	0.65	68.1	59.0	73.5
Combined model	0.755	0.694~0.809	:	67.2	82.0	73.6

IVIM, intravoxel incoherent motion; DKI, diffusion kurtosis imaging; AUC, area under the curve.

D (OR = 1.623, p = 0.046), D* (OR = 0.972, p = 0.002), f (OR = 0.964, p = 0.003), and MK (OR = 0.066, p = 0.011) were independent factors in evaluating the Ki-67 expression status. When D ≤ 0.68 × 10^−3^ mm^2^/s, D* ≥ 31.02 × 10^−3^ mm^2^/s, f ≥ 34.75%, and MK ≥ 0.65, Ki-67 tended to have high expression. The differences in AUCs of D (0.625), D* (0.634), f (0.638), and MK (0.657) were not statistically significant (Z = 0.074~0.705, p = 0.481~0.940). The AUC of the combined model (D + D* + f + MK) was 0.755, being significantly higher than that of each single parameter (Z = 2.770~3.244, p = 0.001~0.006) (**Table 3** and **Figure 3B**).

### Diagnostic Performance of Diffusion-Weighted Imaging-, Intravoxel Incoherent Motion-, and Diffusion Kurtosis Imaging-Derived Parameters in Differentiating Molecular Subtypes of Breast Cancer

**Figure 4:**
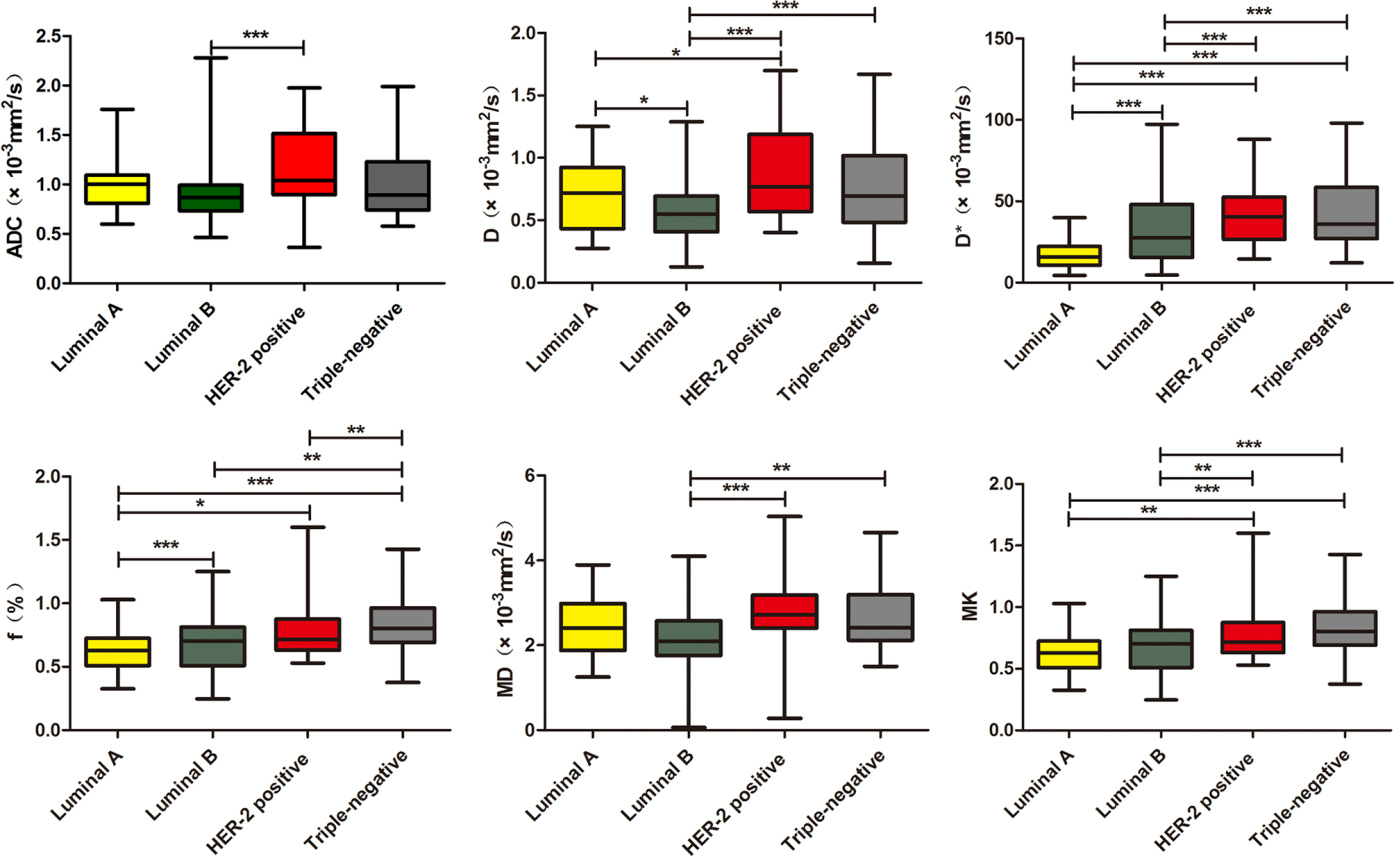
Boxplots of ADC, D, D*, f, MD, and MK in differentiating genotypes of breast cancer. *p < 0.05, **p < 0.01, ***p < 0.001.

The D* and f values in the Luminal A subtype were significantly lower than those of other subtypes (p < 0.05). Luminal A also exhibited decreased D value as compared with the HER-2-positive subtype (p < 0.05). The D value in the Luminal B subtype was significantly lower than that of other subtypes (p < 0.05). The Luminal B subtype exhibited decreased D* and MD values compared with the HER-2-positive and triple-negative subtypes (p < 0.05). The Luminal A/B subtypes (the Luminal subtypes) showed significantly lower D, D*, MD, and MK than the non-Luminal subtypes (p < 0.05). The HER-2-positive subtype demonstrated increased ADC values compared with the Luminal B subtype (p < 0.05). Triple-negative subtypes exhibited increased f value compared with the HER-2-positive and Luminal B subtypes (p < 0.05) (**Table 4** and **Figure 4**).

**Table 4:** Diagnostic performance of DWI, DKI, and IVIM parameters in different genotypes of breast cancer.

Genotypes	n	ADC (×10^−3^ mm^2^/s)	D (×10^−3^ mm^2^/s)	D* (×10^−3^ mm^2^/s)	F (%)	MD (×10^−3^ mm^2^/s)	MK
Luminal A	29	1.01 ± 0.29	0.72 ± 0.30	17.29 ± 8.87	28.88 ± 12.51	2.44 ± 0.73	0.63 ± 0.16
Luminal B	122	0.93 ± 0.32	0.58 ± 0.26	35.23 ± 26.11	40.06 ± 14.13	2.24 ± 0.68	0.68 ± 0.21
HER-2-positive	36	1.16 ± 0.41	0.88 ± 0.38	44.88 ± 24.53	36.79 ± 14.16	2.80 ± 0.80	0.79 ± 0.25
Triple-negative	40	1.02 ± 0.35	0.78 ± 0.36	45.20 ± 23.87	48.75 ± 18.66	2.44 ± 0.76	0.84 ± 0.23
F/χ^2^		4.544	11.101	41.376*	10.585	7.713	8.509
p		0.004	<0.001	<0.001	<0.001	<0.001	<0.001

DWI, diffusion-weighted imaging; DKI, diffusion kurtosis imaging; IVIM, intravoxel incoherent motion.

*Kruskal–Wallis H test.

### Development, Validation, and Diagnostic Efficiency of the Genotype-Prediction Models of Breast Cancer

**Figure 5:**
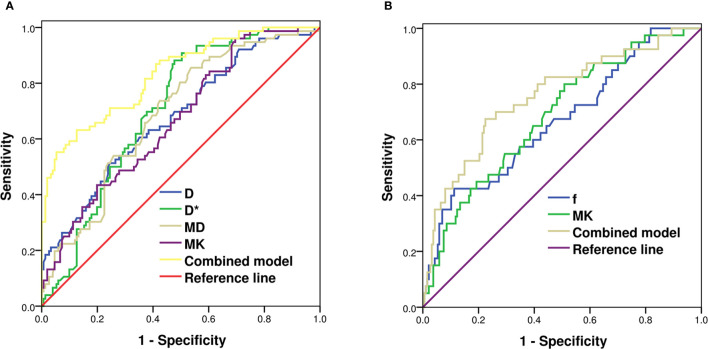
ROC analysis of IVIM and DKI parameters in predicting Luminal subtypes **(A)** and triple-negative subtype of breast cancer **(B)**. ROC, receiver operating characteristic; IVIM, intravoxel incoherent motion; DKI, diffusion kurtosis imaging.

**Figure 6:**
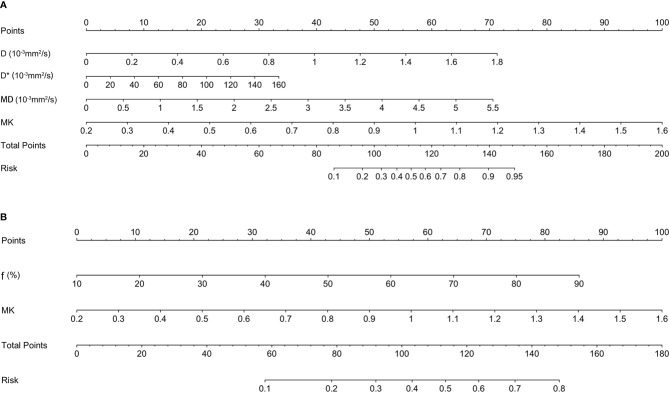
The nomogram for predicting the Luminal subtypes **(A)** and triple-negative subtype of breast cancer **(B)**.

**Figure 7:**
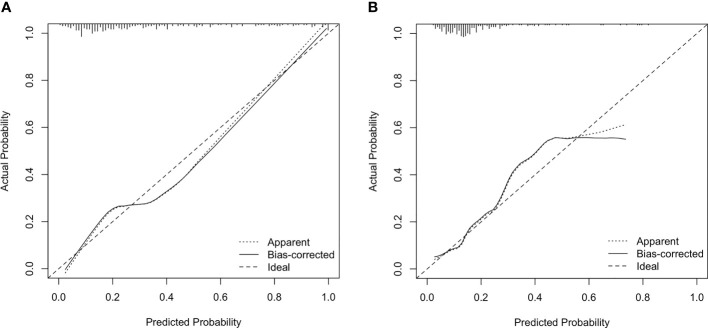
Calibration plot for internal validation of the Luminal subtypes **(A)** and triple-negative subtype **(B)**. The x-axis is the nomogram-predicted probability of genotypes. The y-axis is the actual probability. The dotted line represents an ideal standard curve; the solid line represents the prediction calibration curve of the nomogram. The solid line has a closer fit to the ideal dotted line, which indicates better predictive accuracy of the nomogram (p = 0.22, 0.74).

According to the multivariate logistic regression, D (OR = 21.023, p < 0.001), D* (OR = 1.017, p = 0.025), MD (OR = 1.057, p = 0.001), and MK (OR = 24.745, p < 0.001) were independent factors in identifying the Luminal subtypes from the non-Luminal subtypes. The differences in AUCs were not statistically significant (Z = 0.164~0.826, p = 0.409~0.765). The AUC of the combined model (D + D* + MD + MK) was 0.830, which was significantly higher than that of each single parameter (Z = 3.273~4.440, p < 0.01) (**Table 5** and **Figure 5A**). The nomogram model was thus generated by using these four independent factors as predictors (**Figure 6A**). As shown in the calibration curve, a good agreement was observed between nomogram prediction values and actual findings, and the Hosmer–Lemeshow test showed no significant difference (p = 0.22) (**Figure 7A**).

**Table 5:** Diagnostic efficiency of the IVIM and DKI models for predicting molecular subtypes.

Parameters	AUC	95% CI	Cutoff	Sensitivity (%)	Specificity (%)	Accuracy (%)
**Luminal A/B vs. non-Luminal**						
D	0.676	0.602-0.750	0.66 × 10^−3^ mm^2^/s	60.5	64.9	68.7
D*	0.704	0.637-0.770	24.51 × 10^−3^ mm^2^/s	88.2	52.3	68.9
MD	0.689	0.618-0.759	2.27 × 10^−3^ mm^2^/s	73.7	57.6	67.0
MK	0.666	0.594-0.738	0.537	96.1	30.5	63.3
Combined model	0.830	0.774-0.876	:	73.2	87.4	80.2
**Triple-negative vs. other genotypes**						
f	0.667	0.602-0.728	54.30%	52.5	88.8	66.1
MK	0.686	0.621-0.746	0.68	80.0	49.2	67.2
Combined model	0.756	0.695-0.811	:	67.5	77.5	82.4

IVIM, intravoxel incoherent motion; DKI, diffusion kurtosis imaging; AUC, area under the curve.

f ≥ 54.30% (OR = 1.038, p < 0.001) and MK ≥ 0.68 (OR = 24.745, p = 0.012) were found to be significant predictors of triple-negative subtypes. The AUC of the combined model (f + MK) was significantly higher than that of f (Z = 2.521, p = 0.012), whereas there was no statistically significant difference from that of MK (Z = 1.645, p = 0.100) (**Table 5** and **Figure 5B**). A nomogram was established according to the multivariate logistic regression analysis findings (**Figure 6B**), and there was a good agreement between the bias-corrected curve and the ideal curve as shown in the calibration curve plot and the Hosmer–Lemeshow test (p = 0.74) (**Figure 7B**).

## Discussion

As shown in the results, the functional parameters of DWI, IVIM, and DKI revealed distinct values in different histopathological features and genotypes of breast cancer. For the prognostic factors, D* and MK values were higher in the high- than low-level NPI group. The f, MK, and D* values were higher and the D value was lower in the Ki-67-positive than Ki-67-negative group. In terms of molecular subtypes, the D value of Luminal B was lower than that of other genotypes. D* and f values of Luminal A were lower than those of other genotypes. With the combined model of D, D *, MD, and MK, the diagnostic efficiency of the Luminal subtypes was greatly improved. Compared with those of other genotypes, the f and MK values of TN tumors were higher, and the combination of these two parameters can improve the prediction accuracy of TN tumors.

NPI has been reported as an important value for the prognostic evaluation and formulation of a treatment plan (26). A number of previous studies have shown that the ADC value of DWI, tumor volume doubling time of 3D ultrasound, and contralateral parenchymal enhancement of DCE-MRI could predict NPI (27–29). However, to the best of our knowledge, this study is the first attempt to combine DWI, IVIM, and DKI to predict the NPI of breast cancer. In our study, the IVIM parameter of D* and the DKI parameter of MK are shown as independent predictors in the assessment of NPI. The prediction accuracy of combined D* and MK was higher than that of each single parameter. The pathological basis is that NPI can be used to reflect tumor proliferation and metastasis, which is associated with tumor cell heterogeneity, more microangiogenesis, higher blood volume, and vascularization. A higher grade of NPI manifests higher microperfusion and lower non-Gaussian diffusivity.

Tumor cells with higher Ki-67 expression generally exhibit increasing heterogeneity and complexity of the microstructural level, thereby manifesting higher microperfusion and lower diffusivity (30). In this study, the D, D*, f, and MK values are independent predictors in discriminating the Ki-67 expression status. The AUC of the combined model demonstrated superior diagnostic performance compared with the single parameter, which is consistent with the study of Meng et al. (31, 32). However, in some other studies, it was observed that the expression of Ki-67 has no significant correlation with the D, MD, and MK values (33, 34). We speculated that this discrepancy might be related to the inclusion of lesions, the selection of b-values, and the ROI delineation.

Luminal A breast cancer is defined as a low-proliferation subtype and generally has a favorable prognosis compared with other subtypes. In this study, Luminal A showed the lowest D* and f values, indicating less intratumoral microperfusion. The reason may be that ER/PR expression is associated with the inhibition of angiogenesis, which would reduce perfusion (35). As ER-/PR-positive and higher Ki-67 indices tend to have lower diffusivity and vascularity, we found that Luminal B has the lowest D value. The HER-2-positive subtype demonstrated increased ADC values compared with the Luminal B subtype. The reason might be that HER-2 overexpression exhibits higher angiogenesis, which leads to an increased diffusion (36). The differentiation between the Luminal and non-Luminal tumors is of particular clinical importance since Luminal subtypes are treated with endocrine therapy and may benefit less from cytotoxic chemotherapy. In this study, non-Luminal breast cancer, including HER-2-positive and triple-negative tumors, had a higher D* value than Luminal A/B. It is likely that high vascularity in HER-2-positive and triple-negative tumors may overcome the restricted Gaussian diffusion related to high cellularity, which is in line with findings of Uslu et al. (37). On the other hand, TNBC has a poor response to endocrine or targeted therapy as well as chemotherapy, and the prognosis is worse than that of other subtypes (38). This study found that triple-negative tumors exhibited higher MK values than other subtypes, due to the most complex microstructure. Moreover, we demonstrated that MK could reflect the complexity of the microscopic structures in tissues more accurately, by using sufficiently high b-values of 2,000 s/mm^2^ to eliminate the perfusion effect. In this study, the calibration curve for the combined genotype prediction models indicated that these models had good stability and that the corresponding nomograms could be helpful for visually and interpretatively predicting the genotypes of breast cancer patients.

The present research has some limitations. Firstly, there may be selection bias because of the relatively small sample size and the limited pathological types (most of which were invasive ductal carcinoma). Secondly, no unified standard exists for the option of number and value of b used in IVIM and DKI scanning, and the repeatability of the b-value used in this study requires further verification. Thirdly, we simply calculated the ROI-based mean value of each parameter, which might not be enough to fully reflect the heterogeneity of tumors. An alternative processing method is to extract the whole volume of the lesions and analyze the histogram and texture features of each parameter map, which will be the focus of our future study.

In conclusion, the quantitative parameters of DWI, IVIM, and DKI are correlated with prognostic metrics. D* combined with MK is more valuable for assessing the Nottingham index. ADC, D, D*, f, and MK are valuable for reflecting the KI-67 expression status. The AUC of combined D, D*, MD, and MK could yield robust diagnostic performance for discriminating Luminal A/B from non-Luminal breast cancers. Combined MK and f can facilitate the diagnosis of triple-negative breast cancer. Therefore, this study suggests that the functional parameters of DWI, IVIM, and DKI may reveal clinical potential in the diagnosis of genotypes and prognostic factors and may contribute to opting for an appropriate therapeutic approach in the clinic.

## Data Availability Statement

The raw data supporting the conclusions of this article will be made available by the authors, without undue reservation.

## Ethics Statement

The studies involving human participants were reviewed and approved by the institutional ethics committee of the affiliated hospital of Jining Medical University. The patients/participants provided their written informed consent to participate in this study.

## Author Contributions

WW, XZ, YC, and ZS contributed to the conception and design of the study. LZ and FZ organized the database. ZZ and WD performed the statistical analysis. WW wrote the first draft of the manuscript. WW and ZS contributed to manuscript revision. All authors read and approved the submitted version.

## Funding

This work was supported by Medical and Health Science and Technology Development Project of Shandong Province (Grant number: 202009011151) and the Scientific Research Support Fund for Teachers of Jining Medical University (Grant number: JYFC2019FKJ088).

## Conflict of Interest

Author WD is employed by GE Healthcare.

The remaining authors declare that the research was conducted in the absence of any commercial or financial relationships that could be construed as a potential conflict of interest.

## Publisher’s Note

All claims expressed in this article are solely those of the authors and do not necessarily represent those of their affiliated organizations, or those of the publisher, the editors and the reviewers. Any product that may be evaluated in this article, or claim that may be made by its manufacturer, is not guaranteed or endorsed by the publisher.
